# The Psychosocial Screen for Cancer (PSSCAN): Further validation and normative data

**DOI:** 10.1186/1477-7525-7-16

**Published:** 2009-02-24

**Authors:** Wolfgang Linden, A Andrea Vodermaier, Regina McKenzie, Maria C Barroetavena, Dahyun Yi, Richard Doll

**Affiliations:** 1 Department of Psychology, University of British Columbia, Vancouver, B.C, Canada; 2 British Columbia Cancer Agency, Vancouver, B.C, Canada; 3 Department of Health Care & Epidemiology, University of British Columbia, Vancouver, B.C, Canada; 4 Department of Psychology, Fuller Theological Seminary, Pasadena, CA, USA

## Abstract

**Background:**

We have previously reported on the development of a cancer-specific screening instrument for anxiety and depression (PSSCAN). No information on cut-off scores or their meaning for diagnosis was available when PSSCAN was first described. Needed were additional analyses to recommend empirically justified cut-off scores as well as data norms for healthy adult samples so as to lend meaning to the recommended cut-off scores.

**Methods:**

We computed sensitivity/specificity indices based on a sample of 101 cancer patients who had provided PSSCAN data on anxiety and depression and who had completed another standardized instrument with strong psychometrics. Next, we compared mean scores for four samples with known differences in health status, a healthy community sample (n = 561), a sample of patients with a representative mix of cancer subtypes (n = 570), a more severely ill sample of in-patients with cancer (n = 78), and a community sample with a chronic illness other than cancer (n = 85).

**Results:**

Sensitivity/specificity analyses revealed that an excellent balance of sensitivity/specificity was achievable with 92%/98% respectively for clinical anxiety and 100% and 86% respectively for clinical depression. Newly diagnosed patients with cancer were no more anxious than healthy community controls but showed elevations in depression scores. Both, patients with chronic illness other than cancer and those with longer-standing cancer diagnoses revealed greater levels of distress than newly diagnosed cancer patients or healthy adult controls.

**Conclusion:**

These additional data on criterion validity and community versus patient norms for PSSCAN serve to enhance its utility for clinical practice.

## Background

There is steadily growing interest in routine screening for emotional distress in cancer and other medical patients in order to identify patients who need psychological support most urgently [1]. Emotional distress has been recognized as a critical 6^th ^Vital Sign in medical care [2] thus mandating professional attention. Routine screening of all patients may prevent problem worsening via early intervention, assures equal access to services for all segments of the population, and allows a fair distribution of resources and carries potential for long-term cost savings [3,4]. Furthermore, distress-reducing treatments have been effective only when pre-treatment distress was clearly elevated before treatment initiation [5]. Ignoring this principle translates into a waste of valuable therapy resources that already-strained health care systems can hardly afford. These reasons have led to the development of screening tools for distress.

Large-scale screening requires simple, quick tools with an appropriate balance of brevity and still good psychometrics. Particularly popular is the single item distress thermometer [6] which, however has been criticized for inadequate specificity [7,8] which then requires a referral for additional diagnostics. This inherent weakness of a single-item screening tool makes longer tests a preferred choice. Given that a psychological domain of interest can be tapped satisfactorily with only a few items [7], adding more test items improves the psychometric quality of a tool and permits the assessment of multiple psychological constructs of interest.

In a review of the most frequently used tools for psychosocial distress screening [9], it became apparent that (a) most often measured were anxiety and depression, (b) there was no agreement on the best screening tool, (c) many measures were too long for routine screening, and (d) some tools of interest were copyrighted protected and would have to be purchased for every application.

In light of these observations, we had developed a 21-item instrument (the Psychological Screen for Cancer, PSSCAN; 10) that stands out because of (a) its brevity, (b) its development in the clinical context where it was to become implemented, (c) the scope of the domains being measured, (d) inclusion of both negative and positive aspects of the patients' quality of life (namely level of distress and level of social support), and (e) its non-commercial nature. Note, that after the first article on PSSCAN was published in 2005, we were alerted that the original acronym 'PSCAN' was already copyrighted. Our acronym was then changed to carry one additional 'S' although the full name of the test still is: "Psychosocial Screen for Cancer".

It is the objective of this paper to report additional validation results and normative data for PSSCAN. When PSSCAN was introduced to the literature, the tool's development, indices of reliability, and the establishment of concurrent and construct validity for cancer populations had already been described [10]. PSSCAN assesses anxiety and depression, perceived social support, desired social support, and health-related quality-of-life. It has good psychometrics including high internal consistency (alpha averaging .83, and acceptable test-retest stability over 2 months (averaging r = . 64).

Since then, this tool has been implemented in four Canadian cancer centers [11] and the test developers have received further requests for permission to use PSSCAN from Ireland, the U.S., Japan, Australia, Switzerland, Brazil, Colombia, and Mexico.

Clinicians working with PSSCAN have repeatedly asked for cut-off scores to assist with them with the decision of whether or not a patient had a diagnosable disorder in need of treatment. While researchers can 'bathe in the relative luxury' of statistically treating continuous variables like anxiety as indeed continuous, clinicians are required to make dichotomous decisions about whether or not a given patient has a defined disorder, and will receive a particular form of treatment or further diagnostic services. This is important because health care systems will typically fund psychological treatment only if it is for patients with a diagnosed disorder. No information on cut-off scores and their meaning for diagnosis was available for PSSCAN when it was first published. We now have conducted additional analyses to recommend specific cut-off scores and have also gathered data from healthy, normative adult samples so that both the clinical and healthy norm data can be used to lend meaning to the cut-off scores recommended here.

The specific aims of this paper are to describe the computation of Areas under the Curve (AUC) and resulting sensitivity and specificity indices for the anxiety and depression subscales of PSSCAN. Next it is discussed how sensitivity/specificity information was used to establish empirically-driven cut-off scores. Finally, mean scores and standard deviations on anxiety and depressive symptoms are reported for four samples, representing healthy adults, individuals from the community who have a life-threatening or chronic illness, in-patients with cancer, and a sample of recently diagnosed out-patients with cancer. These comparisons illustrate prevalence rates of anxiety and depression in cancer samples and also place them within the larger context of population norms.

### Study 1: Sensitivity- Specificity Analyses

#### Methods

##### Data collection

Sensitivity and specificity computations were conducted on the data set used originally for concurrent validity testing for PSSCAN, n = 101 [10]. Given that these patients had completed parallel measures of other established anxiety and depression tools which do have empirically justified cutoffs, sensitivity and specificity for PSSCAN cutoffs could be computed. This sample consisted of patients making first contact with the BC Cancer Agency at the Vancouver Center; eligible patients were recruited consecutively by two trained research assistants over a period of one month. The research assistants were physically located in the reception area, were alerted about potentially eligible patients by the receptionist, and then approached patients individually to explain the study, seek consent, and request completion of a test package. All sub-studies were individually approved by the local ethics committee

##### Outcome measures

The questionnaire package consisted of the PSSCAN as described above, the Hospital Anxiety and Depression Scale (HADS; 12) and a social support instrument which, however, was not further investigated here because level of social support is not typically used for making clinical diagnoses, and because no meaningful cut-offs were available for comparison. The HADS is a very frequently used 14-item scale tapping anxiety and depression. Bjelland et al. [13] reviewed the psychometrics of the HADS based on 747 published studies and reported Cronbach's alphas of .68 to .93 for anxiety and .67 to .90 for depression. Factor analyses routinely confirm the underlying 2-factor structure [14-16]. The suggested cutoffs, based on comparisons with structured interviews, to identify subclinical and clinical cases respectively are *8 and above*, and *11 and above*, on the anxiety *and *depression subscales alike [12,13].

##### Statistical analyses

Receiver operating characteristic (ROC) curve analyses were performed for the anxiety and the depression subscales of PSSCAN and the corresponding validated measure namely the HADS anxiety and depression subscales. The resulting ROC curve statistics provide both a visual description of the relationship between PSSCAN data and the criterion indices (HADS anxiety and depression subscales), and allowed the computation of the overall fit statistic, and sensitivity and specificity. A perfect screening tool would explain 100% of the Area Under the Curve (AUC) and would receive a corresponding statistical fit score of 1.0. The AUC is statistically interpreted as describing sensitivity/specificity in percent such that an ideal cutoff would approach 100% on both. Given that it is unlikely that both numbers are exactly the same for any given cutoff score, one needs to decide whether it is more important to have high sensitivity and possibly lower specificity or vice versa. Either decision comes with its own distinct costs. If the test has a very low cutoff, then it is likely to have very high sensitivity and will identify a large number of patients that will then require further, possibly expensive, diagnostic assessments. Given that screening tests are not meant to substitute full clinical diagnoses, a decision to seek higher sensitivity than specificity is considered optimal in that the right kinds of patients are identified with the fewest resources wasted.

With regard to the ROC curve analyses for the two constructs that are measured by PSSCAN and the established criterion measure, namely the HADS subscales, a criterion of 8 or above on the HADS Anxiety Scale was taken as an indication of a subclinical diagnosis and a criterion of 11 or above as a likely clinical diagnosis of elevated anxiety. Likewise, a score of 8 or above on the Depression Subscale of the HADS was taken as a criterion for a subclinical diagnosis and a score of 11 or above as a likely diagnosis of clinical depression [13]. The question here was which cut-off score on the PSSCAN corresponded with these cut-off scores for the HADS subscales.

### Results

**Figure 1:**
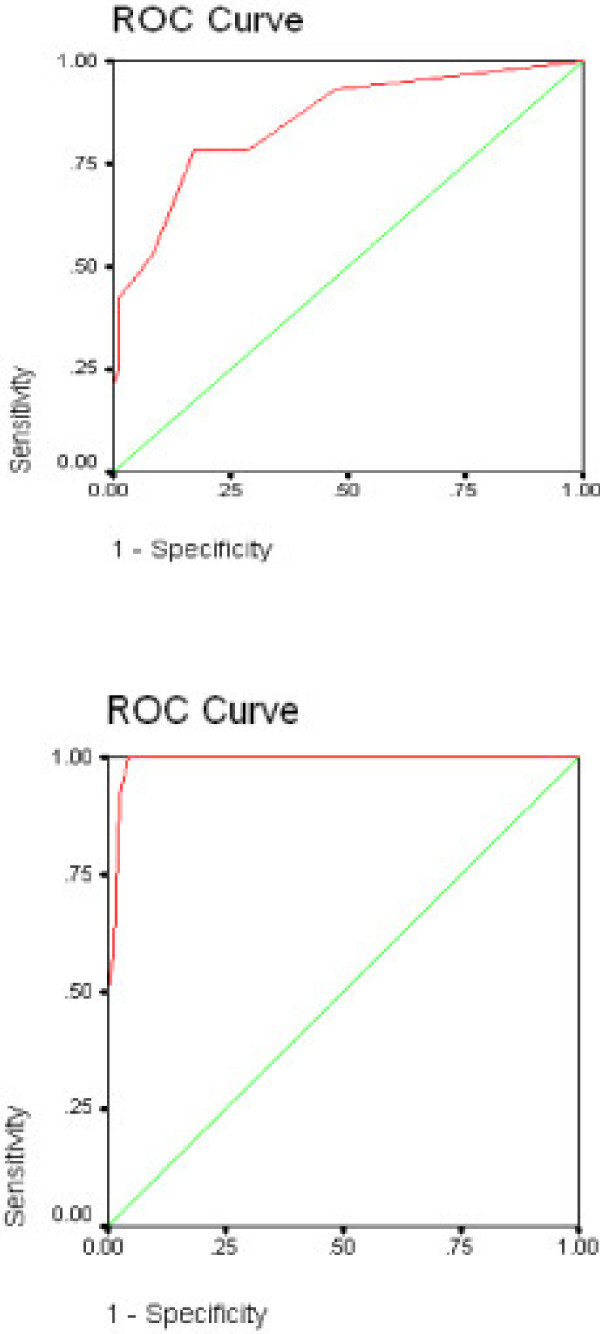
**a and b**. Receiver Operating Curves for the Anxiety Subscale of the PSSCAN with the Anxiety Subscale of the HADS as the Criterion; Fig a: subclinical threshold; Fig b clinical threshold.

**Figure 2:**
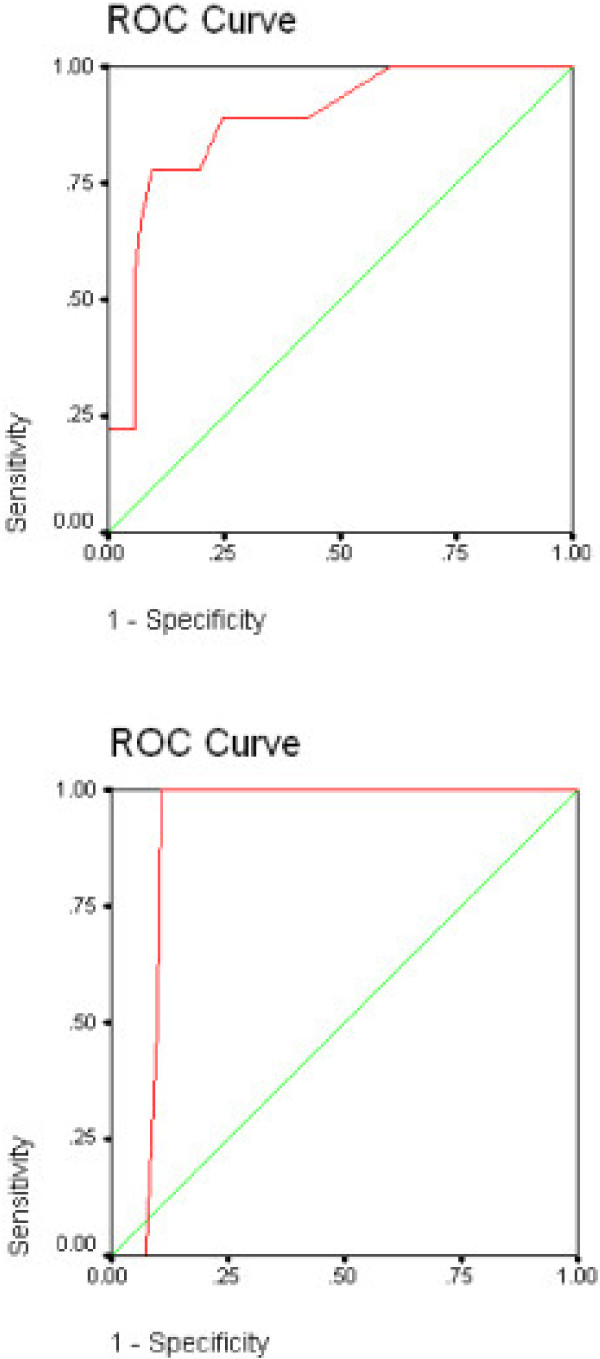
**a and b**. Receiver Operating Curves for the Depression Subscale of the PSSCAN with the Depression Subscale of the HADS as the Criterion; Fig a subclinical threshold; Fig b clinical threshold).

Complete data were available from 101 cancer patients with a mean age of 53 years, composed of 60 women and 41 men. ROC curves are displayed in Figures 1a and 1b, and Figures 2a and 2b; sensitivity/specificity data are shown in Table 1.

**Table 1:** Sensitivity/specificity criteria

**Cutoff (in brackets)**	**Sensitivity**	**Specificity**
**Anxiety**		
Subclinical Disorder (= 8 or >) AUC = 0.85	79%	83%
Clinical Disorder (= 11 or >) AUC = 0.99	92%	98%
**Depression**		
Subclinical Disorder (= 8 or >) AUC = 0.88	89%	76%
Clinical Disorder (= 11 or >) AUC = 0.91	100%	86%

Note. AUC = Area under the Curve

#### Anxiety Subscale

Figure 1a shows the receiver operating characteristic of the PSSCAN anxiety subscale with the HADS anxiety subclinical cutoff score as the criterion. PSSCAN is highly sensitive and specific for screening for anxiety as indicated by an overall Area Under the Curve (AUC) of .85 (P < .001). In addition, Figure 1a also displays the varying sensitivity and specificity percentages depending on which PSSCAN score is used as the cut-point. As the data in Figure 1a indicate, a cut-point of 8 or above is therefore best for identifying mild (subclinical) anxiety and results in a sensitivity of .79 and a specificity of .83.

Using the clinical cutoff of the HADS to identify anxiety disorders resulted in an AUC of .99 (P < .001). The optimal cut-off was 11 or above with a sensitivity of .92 and a specificity of .98 (Figure 1b).

#### Depression Subscale

Figure 2a shows the receiver operating characteristic of the PSSCAN depression subscale with the HADS *subclinical *score as the criterion. An AUC of .88 (p < .001) indicates that the PSSCAN depression subscale is highly sensitive and specific for screening of depression in cancer patients. As the data in Figure 2a indicate, a cut-off point of *8 and greater *results in a sensitivity of .89 and a specificity of .76 to detect depressive symptoms.

Figure 2b shows the ROC curves of the PSSCAN depression subscale with the *clinica*l cutoff of the HADS as the criterion. This resulted in an AUC of .91 (P < .001). The corresponding ideal cutoff on the PSSCAN to detect major depressive disorders was *11 and greater *with a sensitivity of 1.00 and a specificity of .86.

### Study 2: Criterion Validation and Population norms via Comparison of Patient versus Non-patient Groups

#### Methods

##### Participants and accrual of samples

Criterion validity was tested by comparing four samples that were known to differ in health status.

Sample 1 was the large sample (n = 570) of cancer patients described in the original manuscript [10]. Sample 2 was a small in-patient sample of cancer patients, and Samples 3 and 4 were community samples. Sample 2 was obtained by collecting PSSCAN information from patients on an inpatient ward in the local cancer center. This inpatient ward typically serves roughly equal portions of two kinds of patients, namely one group with fairly advanced cancer who will likely move from the acute cancer ward to a palliative care environment, and another group that requires extensive tests and/or treatment; these latter patients come from outlying communities and could not make themselves available on a daily basis for treatments or lengthy assessments during the day, and then return home at night. A research assistant spent one month approaching all patients on the ward by scanning charts for newly arrived patients. A total of 78 participants were thus accumulated for sample 2, which is characterized by an established diagnosis of cancer and typically advanced disease with unknown or poor prognosis. This sample had a mean age of 56.9 years, representing 39 women and 39 men.

Samples 3 and 4: In order to access a fairly representative sample of adults living in the community, two research assistants approached commuters waiting for a car ferry. This ferry has a shuttle function and crosses a local river in five-minute intervals. Given that the ferry capacity is routinely insufficient for the amount of traffic, commuters typically spend between 15 and 60 minutes waiting for the ferry, sitting in their cars on a public road, with little to do. Depending on the time of day this ferry transports people on their way to and from work, or shoppers and casual travelers between two communities. The research assistants moved from car to car, introduced themselves, revealed photo IDs identifying them as research assistants of the local university, explained the study to participants, and obtained written consent to participate. Over 90% of all individuals asked to participate, did so and received a set of two different-colored ballpoint pens with the logo of the university as a gift in exchange for their time. In addition to completing the PSSCAN, they also indicated their age and gender, and responded to the question of whether or not they had a chronic illness. Individuals reporting a positive diagnosis of cancer were excluded from these community samples. Chronic illness was defined as having heart disease, arthritis, diabetes, or an autoimmune disease, or any other disease of similar severity (participants provided this information in an open response form). A minimum age threshold of 40 years of age was set for participation in order to increase the probability that the resulting sample was similar in age to typical cancer populations which usually have a mean age between 50 and 60 years. No upper age limit was set. The resulting sample was on average 53.6 years old and consisted of 358 women and 394 men. Complete data were available for 561 participants who declared themselves to be healthy, and another 85 participants who reported to have a chronic illness.

This sample of convenience represents a wide range of ages, both sexes, as well as people of varying socio- economic strata given that there is only one ferry system in this location for people of all income levels. This data collection process provided samples 3 and 4, one healthy, the other one with a chronic illness.

## Results

Means and standard deviations for all four samples are displayed in Table 2 allowing the comparison of anxiety and depression scores for four groups of people, one cancer outpatients, another one a group of inpatients with more advanced cancer, one large group of healthy community members, and another comparison group of community members with a chronic disease other than cancer.

**Table 2:** PSSCAN means (and SD) for anxiety and depressive symptoms in four comparison samples, and effect size *d *for the differences of all paired sample comparisons

	Sample 1 Cancer Out-patients, N = 570	Sample 2 Cancer In-patients N = 78	Sample 3 Community sample with chronic illness N = 85	Sample 4 Healthy Community sample N = 561
Anxiety	8.2 (4.2)	10.9 (5.1)	10.2 (4.9)	7.7 (3.3)
	1 vs 2: d = -.57*	*2 vs 3: d = .14*	3 vs 4: d = .81*	
	1 vs 3: d = -.43*	2 vs 4: d = .76*		
	*1 vs 4: d = .10*			
Depression	8.2 (5.1)	9.8 (4.6)	9.4 (4.9)	7.2 (2.7)
	1 vs 2: d = -.33*	*2 vs 3: d = .08*	3 vs 4: d = .58*	
	1 vs 3: d = -.24*	2 vs 4: d = .70*		
	1 vs 4: d = .26*			

* = p < .01 on t-test

Inferential tests were conducted by first computing effect sizes (Cohen's d) and subsequent extraction of critical thresholds from power tables. Given that we conducted multiple pair-wise tests (five tests per outcome variable), we used Bonferroni corrections and set the critical p-value at p = .01 for 99% power [17]. The between-group differences for each of the five comparisons per variable are displayed as effect sizes in Table 2.

As the data in Table 2 reveal, recently diagnosed out-patients with cancer reported less anxiety than in-patients with cancer, and less anxiety than community-living patients with other chronic illnesses; they were no more or less anxious than a healthy community comparison group. The in-patients with cancer reported more anxiety than the healthy community sample but not more than the community-living sample with a chronic illness other than cancer. Lastly, the healthy community sample reported less anxiety than the ill community sample.

With respect to depressive symptoms, the results were similar. Recently diagnosed out-patients with cancer reported fewer depressive symptoms than cancer in-patients, reported as many depressive symptoms as community-living patients with other chronic illnesses, and they were more depressed than the healthy community comparison group. The in-patients with cancer reported more depressive symptoms than the healthy community sample but not more than the community sample of people with non-cancer illnesses. Lastly, the healthy community sample reported fewer depressive symptoms than the ill community sample.

## Discussion

The first objective of this research was to identify cut-off points that represented the best balance of sensitivity and specificity for the anxiety and depression subscales of PSSCAN and these were compared against a similar, well established measure that had been validated against gold standard definitions of anxiety and depression. These computations revealed that a score of eight and above on the anxiety and the depression subscales respectively were associated with a high sensitivity and specificity for the detection of anxiety and depressive *symptoms*. A cut off score of 11 and above for anxiety and depression scales respectively possessed even higher sensitivity and specificity of the two PSSCAN subscales in their ability to detect *clinical *levels of anxiety and depression. These findings suggest that PSSCAN, despite its brevity, offers sufficient sensitivity and specificity to be useful not only for initial screening but for the establishment of a working diagnosis that justifies a referral to a mental health professional.

The second objective was to place these cut-off scores in the context of norms for different populations. This comparison allowed two main conclusions. First of all, review of the percentile scores for sample 1 (displayed in table 3) that 16% percent of patients will be declared clinically anxious using a PSSCAN cut-off score of 11 and above, and 18% will be identified as likely clinically depressed by using a depression cut-off score of 11 and above.

**Table 3:** Percentiles for norming (Sample 1, n = 570 cancer patients)

**Anxiety**		**Depression**		**Distress**		**Suicidality**	
**Score**	**%**	**Score**	**%**	**Score**	**%**	**Score**	**%**
5	31.8	5	36.1	10	22.7	Not at All	91.7
6	46.2	6	48.7	12	42.3	A Little Bit	97.0
7	58.2	7	59.6	14	55.7	Moderately So	98.1
8	68.1	8	67.3	16	65.9	Quite a Bit	98.8
9	75.5	9	71.9	18	73.4	Very Much So	100
10	81.4	10	77.4	20	78.6		
11	83.8	11	81.8	22	84.8		
12	86.6	12	85.6	24	87.0		
13	89.6	13	88.5	26	89.2		
14	91.6	14	90.8	28	92.1		
15	92.9	15	93.4	30	93.9		
16	94.9	16	95.5	32	95.9		
17	96.2	17	97.0	34	97.4		
18	97.2	18	98.0	36	98.4		
19	98.5	19	98.4	38	98.9		
20	99.0	20	98.6	40	99.1		
21	99.4	21	98.8	46	99.6		
24	100	22	99.2	48	100		
25	100	23	99.5	50	100		
		24	99.8				
		25	100				

Secondly, comparison of the four samples with each other revealed that the sample of recently diagnosed cancer patients was not more anxious than the healthy community group but patients did have higher depression scores than healthy individuals. Recently diagnosed cancer patients reported levels of anxiety and depression similar to the sample of adults drawn from the community who reported having a chronic disease other than cancer. Cancer inpatients also tended to be more anxious and depressed than other comparison groups. Overall, our data suggest that the prevalence of elevated anxiety and depressive symptoms as assessed by PSSCAN are relatively low compared to a number of other studies that attempted to determine population prevalence of negative mood [18,19].

In terms of clinical implications, we posit that the suggested cut-off scores are empirically justified decision-making points for everyday clinical practice. Clinicians can use the higher or lower cut-offs for subclinical and clinical levels of distress respectively to determine which patients should be referred for further diagnosis and treatment. It also appears that the great majority of newly diagnosed cancer patients do not present with anxiety and depressive disorders and that patient counseling services and local service providers are not likely to get overwhelmed with a need for clinical service when distress screening is routinely conducted (see prevalence rates in table 3).

There are, of course, limitations to this work. In particular, the comparison of mean scores for the different samples should be undertaken with some caution given that we are comparing groups of people who were recruited by different means; and for many of them we have limited amounts of information. For example, relying on self-report about presence of a chronic illness is admittedly crude although we don't doubt the veracity of self-report. Also, comparisons of the two smaller samples are predictably less trustworthy and probably more difficult to replicate than the comparisons of the much larger samples. We do not know whether participants differed in economic status or ethnic origin. Given that to the best of our knowledge no such recruiting method has been used previously, we can only speculate about comparability. In both instances, respondents were free to make their own choices; roughly 90% of eligible participants in both settings participated, and we used an age cutoff as a selection strategy in order to achieve a roughly age-matched control sample. Furthermore, the situations were similar in that respondents were in a waiting situation, seated with reasonable comfort, and questionnaire completion might actually have been a welcome distraction.

The reader may be tempted to ask why one should not use the HADS instead of PSSCAN given that the sensitivity/specificity of the tool had been compared with that of the HADS in the first place. There are two reasons for continuing work on the PSSCAN: [a] The HADS is a copyrighted instrument that needs to be purchased whereas PSSCAN is free and placed in an open access journal. [b] The second major difference is that the HADS measures only two constructs, namely anxiety and depression. PSSCAN on the other hand measures five psychological constructs, namely perceived social support, desired social support, and quality of life in addition to tapping into the anxiety and depression. It represents a more comprehensive measure of psychological constructs of interest for Psycho-Oncology and other chronic diseases.

In summary, the additional data reported here regarding validity and norms for PSSCAN provide additional support for the utility of PSSCAN in everyday clinical practice.

## Abbreviations

AUC: Area under the Curve.

## Competing interests

The authors declare that they have no competing interests.

## Authors' contributions

WL contributed to design, the statistical analyses, and was the primary manuscript author; AV contributed to the statistical analyses and was secondary author, RM contributed to design and data collection; MCB contributed to the design, DY assisted with data collection and statistical analysis, RD contributed to the design and writing of the manuscript
